# Comparative Analysis of Chemical Composition andAntibacterial and Anti-Inflammatory Activities of theEssential Oils from *Chrysanthemum morifolium* ofDifferent Flowering Stages and Different Parts

**DOI:** 10.1155/2022/5954963

**Published:** 2022-06-06

**Authors:** Xia-Jin Liu, Yi Li, Shu-Lan Su, Dan-Dan Wei, Hui Yan, Sheng Guo, Er-Xin Shang, Xiao-Dong Sun, Jin-Ao Duan

**Affiliations:** ^1^National and Local Collaborative Engineering Center of Chinese Medicinal Resources Industrialization and Formulae Innovative Medicine, Jiangsu Collaborative Innovation Center of Chinese Medicinal Resources Industrialization, Jiangsu Key Laboratory for High Technology Research of TCM Formulae, Nanjing University of Chinese Medicine, Nanjing 210023, China; ^2^Jiangsu Hexiang Juhai Modern Agricultural Industrialization Co., Ltd., Yancheng 224335, China

## Abstract

The inflorescence of *Chrysanthemum morifolium* Ramat., a well-known traditional Chinese herb, has been proved to have a certain inhibitory effect on some bacteria; however, its main components and acne bacteria inhibition effect remain to be elucidated. In this study, GC-MS was used to analyze the components of different flowering stages and different parts and to study the inhibitory effects of six essential oils on *S. aureus* and *P. acnes* and their alleviating effects on THP-1 cell inflammation. GC-MS combined with relative retention index method analyzed results stated that the 5 samples of *C. morifolium* to detect the 124 kinds of volatile components, including (*E*)-tibetin spiroether, are first detected in the volatile oil of the *C. morifolium*, and the content of (*E*)-tibetin spiroether is higher in immature inflorescence of *C. morifolium* and decreases as it extends its flowering period. Furthermore, the research results of inhibiting common acne-causing bacteria showed that the bacteriostatic effect of essential oils from JH at different flowering stages was better than that from JM and TJ, while the bacteriostatic effect of essential oil from stem and leaf of *C. morifolium* (SLC) at different parts was better than the roots of *C. morifolium* (RC). Finally, it was proved that the essential oil from SLC and *C. morifolium* could alleviate the inflammation of THP-1 cells induced by *P. acnes*. In conclusion, the antibacterial and anti-inflammatory effects of *C. morifolium* essential oil may be related to heterospiroolefins compounds, and the antibacterial activity decreases with the prolongation of flowering stage. It was suggested that volatile oil from *C. morifolium* and SLC could be used as effective components of antibacterial and anti-inflammatory cosmetics.

## 1. Introduction

The *Chrysanthemum* genus (*Chrysanthemum morifolium* Ramat.) belongs to the Compositae family, mainly distributed in temperate regions, and has been widely used as medicinal, edible, and ornamental plants for thousands of years [1, 2]. There are more than 200 species and varieties of Compositae plants, including *Chrysanthemum indicum*, *Chrysanthemum trifurcatum* (Desf.), and *Chrysanthemum japonense*, all of which have remarkable pharmacological activity [3–5]. The capitulum of *Chrysanthemum morifolium* Ramat. was used as Hangbaiju in China and is rich in volatile oils, flavonoids, phenolic acids, and polysaccharides. Recently, pharmacological studies have revealed that it has broad bioactivities, such as anti-inflammatory, antitumor, relieving diabetes, and antioxidative damage activities [6, 7]. It has been proved that chrysanthemum extract can improve the specific skin inflammation in mice and has the potential to treat various immune-related skin diseases [8]. Studies have shown that adding 0.5% water extract of chrysanthemum to beauty cream can effectively reduce melanin levels, indicating that chrysanthemum water extract can be used as a natural whitening agent for functional cosmetics [9]. In addition, creams containing chrysanthemums were more effective than placebo in reducing the erythema of rosacea, suggesting that chrysanthemums and their nonmedicinal parts with similar activity have a broad application prospect in cosmetics [10, 11].

Remarkable differences existed in the active components of chrysanthemum at different flowering stages and in different parts, and the active functions were related to them to some extent [12]. Comparing the chemical constituents and antioxidant activities of *Chrysanthemum* (*Chrysanthemum boreale* Makino) essential oils at different flowering stages, it was found that the antioxidant activity was the highest at full flowering stage, followed by vegetative stage and preflowering stage, which was related to the change of eugenol [13]. Previous studies have been conducted on the chemical composition and antimicrobial activity of Compositae plants, such as *Chrysanthemum indicum* L., *Chrysanthemum trifurcatum*, *Chrysanthemum boreale*, and *Chrysanthemum coronarium* [14, 15]. However, no systematic comparative study was made on the constituents of volatile oil in different flowering stages and different parts of *Chrysanthemum morifolium* Ramat. Most of the researches on the bacteriostatic effect of chrysanthemum essential oil focus on the fields of bacteriostatic agent, disinfectant and substitute for antibiotics, and so on, and there are few researches on bacteria in the fields of daily chemical [16].

In the present study, not only the composition analysis and antibacterial evaluation of *C. morifolium* essential oil from different flowering stages and different medicinal parts were carried out, but also evaluated for alleviating inflammation of THP-1 cells caused by *P. acnes*. Moreover, in order to develop an anti-inflammatory and acne-removing skin care product and expand the application of *C. morifolium* essential oil in daily chemical products, this paper selected *Propionibacterium acnes* and *Staphylococcus aureus*, which are prone to occur on the face and cause acne, to carry out anti-inflammatory and bacteriostasis experiments, providing a certain support for the preparation of anti-inflammatory and acne-removing agents.

## 2. Experimental Section

### 2.1. Plant Materials

Ju-Mi (JM), Tai-Ju (TJ), and Ju-Hua (JH) are three kinds of processed *Chrysanthemum morifolium* flower heads harvested at different flowering stages. The stems-leaves (SLC) and roots (RC) of chrysanthemum are different parts of medicinal chrysanthemum. Dry JM, TJ, and JH and Fresh SLC, RC of *Chrysanthemum morifolium* were collected from Shenyang in Jiangsu Province in November 2019. The air-dried SLC, RC were obtained directly by air-drying method. All the samples were authenticated by Professor Jin-ao Duan as the JM, TJ, JH, stems-leaves, and roots of *C. morifolium*; the voucher specimens were deposited at the Herbarium in Jiangsu Key Laboratory for TCM Formulae Research, Nanjing University of Chinese Medicine, China.

### 2.2. Essential Oils Extraction

JM, TJ, JH, SLC, and RC of the same weight (200 g each) were weighed and placed in the volatile oil extractor, respectively, and extracted by hydrodistillation extraction (HDE) method for 8 h. The essential oils obtained were dried with anhydrous sodium sulphate and stored in the dark at −30°C for subsequent experiments. Yields based on dry weight of the samples were calculated.

### 2.3. GC-MS Analyses

The essential oils were analyzed using a Clarus 680 GC-MS system (Perkin Elmer, USA) equipped with a 30 m × 0.25 mm × 0.25 *μ*m Elite-5MS column (5% diphenyl 1: 95% dimethylpolysiloxane). Essential oils' components were qualitatively and quantitatively determined using high-resolution gas chromatography/quadrupole tandem time-of-flight mass spectrometry (AxION® iQT™ GC/MS/MS). Because it is equipped with a cold EI source, it can effectively generate molecular ion peaks, improving the accurate characterization of compounds. The heating procedure was to keep the initial temperature at 70°C for 2 min, increasing to 130°C at a rate of 10°C/min; then programmed temperature was raised to 150°C at a rate of 1°C/min, increasing to 210°C at 4°C/min and finally held isothermal for 3 min. The solvent delay time was 4 min. The carrier gas was helium at a flow rate of 1.0 mL/min; split ratio was 10 : 1. Injector temperature was 260°C; diluted samples (1.0 *μ*l, 1/100 in hexane) were injected manually. MS parameters were as follows: EI mode, ion source temperature: 180°C; and scan range (*m*/*z*): 50–620.

### 2.4. Identification and Quantification of the Essential Oils' Components

Mass spectrometry and chromatogram analysis were performed using AxION eCipher software, which is compatible with Perkin Elmer Clarus 680 GC-MS. The components were preliminarily matched by comparison of their mass spectra with those of NIST 2.0 library data of the GC-MS system. The relative retention index was calculated based on the same series of *n*-alkanes (C7–C40), and the volatile components in essential oils were further confirmed by combining with published relevant literature.

### 2.5. Antibacterial Activity

#### 2.5.1. Bacterial Strains

Six essential oils were tested against 2 types of Gram-positive bacteria commonly found in cosmetics, of which the *Staphylococcus aureus* (ATCC25923) were donated by the Shanghai Medical Research Institute, and *Propionibacterium acacia* (ATCC6919) were purchased from the Preservation Center of the Microbial Species in Guangdong, China. The strains were stored at −80°C in Microbiology Laboratory, College of College of Pharmacy, Nanjing University of Chinese Medicine. *S. aureus* and *P. acnes* were preserved nutrient agar (Sigma) at 37°C, prepared overnight cultures in MH broth (Mueller–Hinton) and BHI (Brain-Heart Infusion) broth, and adjusted to approximately 10^8^ cfu/mL, respectively.

#### 2.5.2. Microdilution Method

The antibacterial activity of six essential oils was evaluated by broth microdilution method. Anhydrous ethanol was used as solvent to prepare 1000 mg/mL mother liquor of essential oil; all tests were performed in MH broth and BHI broth. *S. aureus* was incubated overnight in a constant temperature chamber (12 h, 37°C, 5% CO_2_), while *P. acnes* was cultured in an anaerobic chamber (48 h, 37°C, 10% CO_2_, 10% H_2_, 80% N_2_), and the final concentration of each strain was adjusted to 3 × 10^4^ CFU/mL. Serial doubling dilutions of the oils were prepared in a 96-well microtiter plate with concentrations ranging from 0.195 to 100.00 mg/mL, and then 100 *μ*L bacterial solution was successively added and incubated in an incubator for 12 h or 24 h to observe whether the bacterial solution was turbidized. If there was no turbidized precipitation, the growth activity of bacteria was inhibited; the concentration of essential oil was MIC at this point. MIC was defined as the minimum inhibitory concentration of essential oil against bacteria at which microorganisms do not demonstrate visible growth [17]. Ethanol used for solubilizing the oil was used as solvent control, and the blank control was used without any added drug.

Control was performed for every assay and showed no inhibition of the microbial growth, except blank control. Penicillin and erythromycin lactobionate were used as positive antibiotics for *S. aureus* and *P. acnes*, respectively [8]. All inhibition measurements were repeated in triplicate.

### 2.6. Effects of JH and SLC Essential Oils on THP-1 Inflammation

#### 2.6.1. Cell Culture of THP-1 Cells

A human monocytic cell line THP-1 cell was obtained from the Shanghai Zhongqiao Xinzhou Biotechnology Co., Ltd., and grown in RPMI-1640 medium accompanied with 10% fetal bovine serum, 1% antibiotics, and *β*-mercaptoethanol 50 *μ*mol/L. The cells were maintained under the environment of 37°C temperature, 5% CO_2_, and 95% of air.

#### 2.6.2. Cytotoxicity Assay

The CCK8 assay was used to assess the THP-1 cell viability and cell proliferation under the action of JH and SLC essential oils. Cells at exponential growth stage were inoculated in 96-well culture plates and the cell density was adjusted to about 9 × 10^4^ cells/mL by RPMI-1640 medium. JH and SLC essential oils with different mass concentrations (final mass concentrations 6.25, 12.5, 25, 50, and 100 mg/mL) were added and incubated at 37°C for 36 h. After centrifugation for 5 min at 800 rmp, the medium was discarded and 100 *μ*L 10% CCK8 solution was added. Absorbance values were measured at 450 nm using a 96-well microplate reader after further incubation for 3.5 h. All experiments were performed in triplicate, and cell viability was expressed as percent change relative to vehicle-untreated control cells.

#### 2.6.3. *P. acnes* Culture

*P. acnes* (ATCC6919) was cultured to logarithmic growth stage and its OD600 was adjusted to 1.0. The logarithmic bacterial culture was centrifuged at 4000 rpm at 4°C for 15 min, and the culture supernatant was discarded. The bacterial precipitation was placed in 100 mL phosphate buffer salt (PBS), washed three times, and finally suspended in 10 mL PBS. The bacterial PBS suspension was placed at 80°C for 30 min to achieve heat inactivation reaction. The thermally inactivated *P. acnes* suspension was stored at 4°C until use.

#### 2.6.4. ELISA for Cytokine Measurements

THP-1 cells were inoculated into 96-well plates containing complete culture medium, and the concentration of cells was adjusted to 1 × 10^5^ cells/mL. After 24 h of culture, fresh culture medium containing JH and SLC essential oil (50 mg/L) was added again for 4 h, respectively. The cells were treated with heat-killed *P. acnes* co-cultured for 8 h. Centrifuged at 3000 rpm/min for 20 min, the supernatant was carefully collected into the ribozyme-free EP. The level of IL-1*β* in cell supernatant was determined by ELISA kit [18].

## 3. Results and Discussion

### 3.1. Percentage Extractive Yield of Essential Oils

By hydrodistillation of air-dried JM, TJ, JH, SLC, and RC, the yields of the six essential oils obtained are 0.44%, 0.35%, 0.21%, 0.16%, and 0.12% (all the above proportions are the quality of essential oil/weight of dried medicinal materials), respectively, with the highest yield for the JM oil (0.44%) and the lowest yield for the RC oil (0.025%) among the five different states of the medicinal plant *Chrysanthemum morifolium*. The afforded JM, TJ, JH, and SLC essential oils have light yellow and pleasant aroma at room temperature, while the essential oils obtained by RC distillation are orange-red and have a pleasant woody fragrance.

### 3.2. Chemical Composition of the Essential Oils

**Figure 1:**
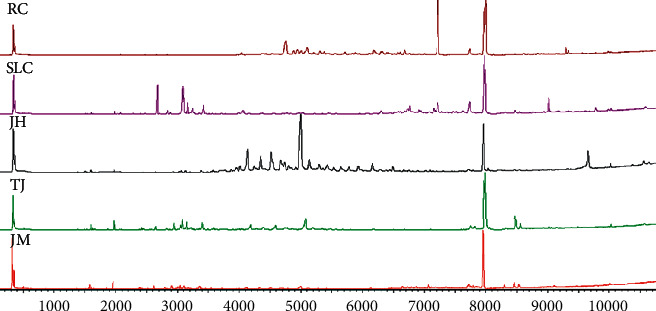
Total ion current of essential oils from JM, TJ, JH, SLC, and RC.

Qualitative and quantitative analytical results by GC and GC/MS are shown in Table 1. And the typical total ion current (TIC) of six essential oil samples of *C. morifolium* is shown in Figure 1. The identification of the oil components was based on the comparison of their mass spectra and retention indices (RI) with those reported in the literature [19–23]. The chromatographic analysis resulted in the obtainment of 124 components and identification of 114 components, representing 89.25%∼96.70% of the six essential oils.

#### 3.2.1. Comparison of *Chrysanthemum* Essential Oils at Different Flowering Stages

As presented in Table 1, compared with JM, TJ, and JH from three different flowering stages, the contents of heterospiroolefins, sesquiterpene hydrocarbons, and hydrocarbon in the volatile oil decreased gradually with the growth of flowering stage. Among them, the contents of heterospiroolefins decreased significantly from 44.81% in JM essential oil to7.27% in JH. Furthermore, the average contents of oxygenated sesquiterpenes in three different flowering stages of *C. morifolium* were ranked in the order of JH > TJ > JM, from the highest to the lowest, and accounted for approximately 73.24%, 26.91%, and 12.47% of the total essential oils, respectively. According to the above analysis, the chemical composition and content of the volatile oil of chrysanthemum are obviously different in different growth stages of chrysanthemum, which should be carefully treated in the development of medicine and health care products. Additionally, the highest content of sesquiterpenoids in TJ (6.44%) and JH (28.62%) was *α*-cadinol, while the highest content of sesquiterpenoids in JM is *β*-sesquiphellandrene, with a content of 2.21%. Sesquiterpenoids play an extremely important role in living organisms and have a variety of pharmacological effects, which laid a certain foundation for the development and application of chrysanthemum essential oil in cosmetics and medicine.

#### 3.2.2. Comparison of *Chrysanthemum* Essential Oils at Different Parts

Remarkable diﬀerences were also observed between the flowerhead, roots, leaves, and stems of *C. morifolium*. In terms of the number of compounds identified, the number of compounds identified from JH, SLC, and RC was 77, 71, and 57, respectively. The number of JH compounds is much higher than that of RC compounds. JH (96.70%), SLC (94.28), and RC (89.25%) also had certain differences for the relative content of essential oils obtained. The content of sesquiterpenes in SLC (27.76%) was 5.7 and 64 times that of JH and RC, respectively. The major compound of sesquiterpenes in SLC was caryophyllene (8.84%), while the main compound of sesquiterpenes in JH and RC was iso-caryophyllene (1.77%) and (*E*)-*β*-farnesene (0.11%), respectively. Natural bicyclic sesquiterpenes, caryophyllene are present in a large number of plants worldwide. Caryophyllene possesses significant anticancer and analgesic activity, and it can improve the systemic inflammation and oxidative status of arthritic rats [24]. In addition, caryophyllene is also approved as food additive, taste enhancer, and flavoring agent and termed as a phytocannabinoid. Present results indicate that the most plentiful constituent of flowerheads essential oils from *C. morifolium* was *α*-cadinol (28.62%). But in the SLC and RC oils, (*E*)-tibetin spiroether (20.53%) and 2-trans, 6-trans-farnesyl acetate (32.00%) were the predominant compound, respectively. However, the essential oils from all parts of *C. morifolium* were rich in terpenoid components, which is consistent with the research results of most researchers. The results clearly revealed that the JM, TJ, and SLC oils were dominated by heterospiroolefins with the major compound being (*E*)-tibetin spiroether, but JH oil was dominated by oxygenated sesquiterpenes with the major constituent was *α*-cadinol. (*E*)-Tibetin spiroether was first reported in *Artemisia roxburghiana* Besser essential oil [25] but never appeared in *Chrysanthemum morifolium* essential oil. Due to its high proportion in JM, TJ, and SLC essential oils, the chemical composition and pharmacological action of (*E*)-tibetin spiroether are worth our attention.

### 3.3. Antimicrobial Activity

The antibacterial activity of *C. morifolium* essential oils against 2 types of Gram-positive bacteria (*S. aureus* and *P. acnes*) was evaluated by the microdilution method to determinate the minimum inhibitory concentration (MIC) (Table 2).

To varying extents, the six essential oils (JM, TJ, JH, SLC, and RC) inhibited the growth of two bacteria commonly found in cosmetics according to the results given in Table 2. The minimum inhibitory concentrations (MIC) of JM, TJ, and JH volatile oils against *S. aureus* and *P. acnes* were 10 mg/mL and 25 mg/mL, respectively. By comparing the oils from different parts, the JH oil was found to be the most effective, followed by SLC and RC for *S. aureus*, on which JH oil shows twice and five times inhibitory effect that of SLC and RC oil. The content of terpenoids in JH essential oil reached 78.74%, which was much higher than SLC (40.13%) and RC (41.82%) oils. The above results are consistent with literature reports, indicating that terpenoids in essential oils have significant antimicrobial activity, and terpenoids can cause bacterial death by destroying the integrity of cell membranes or making bacteria lose part of their functions [26]. For SLC essential oil, its inhibitory effect on *S. aureus* was lower than that of JH, and its MIC value was 20 mg/mL. The roots' oil showed the lowest activity against all strains, and its MIC value was 50 mg/mL. This may be due to the fact that the main metabolites of the esters fraction in RC volatile oil were 2-trans, 6-trans farnesyl acetate. The main components of other five oils are (*E*)-tibetin spiroether, except the JH oil with *α*-cadinol containing oxygen sesquiterpenes. Since these five essential oils have similar antibacterial activities against these two bacteria, we can speculate that *α*-cadinol and (*E*)-tibetin spiroether may have some similar antibacterial activities.

To the best of our knowledge, this is the first time that chrysanthemum essential oil has been studied against *P. acnes* is a common, chronic skin disease that arises in the hair follicle and often involves inflammation [27]. It has been reported that *P. acnes* is thought to trigger inflammatory responses and lead to subclinical and inflammatory acne lesions [28]. In the treatment of acne, erythromycin, clindamycin, and other antibiotic preparations are often used, easy to cause bacterial resistance [29], so it is necessary to study the inhibitory effect of natural plants on the growth of *P. acnes*.

### 3.4. Effects of JH and SLC Essential Oils on THP-1 Inflammation

#### 3.4.1. Effect of JH and SLC Essential Oils on Cytotoxicity of THP-1

According to the previous study, JH and SLC essential oils in different parts had better antibacterial effect, so the anti-inflammatory effect of JH and SLC essential oils was tested.

**Figure 2:**
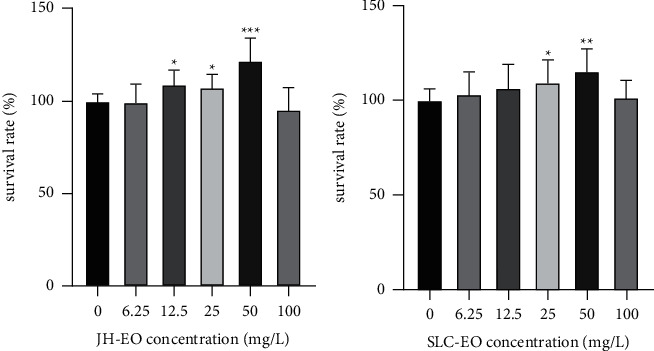
Survival rate of THP-1 cells treated by JH and SLC essential oils (EO). The data are presented as the mean ± SD. ^*∗*^*p* < 0.01, ^*∗∗*^*p* < 0.01, and ^*∗∗∗*^*p* < 0.001 versus the control group, by one-way ANOVA with Tukey's test.

To determine the cytotoxicity, THP-1 cells treated with different concentrations of JH and SLC essential oils (100, 50, 25, 12.5, and 6.25 mg/L) for 36 h were analyzed by an established CCK-8 assay. The effects of JH and SLC essential oils at different concentrations on the survival rate of THP-1 are shown in Figure 2. JH and SLC essential oils have no toxic effects on THP-1 cells within the range of the measured mass concentration (6.26 mg/L–100 mg/L), which fully demonstrates the safety of JH and SLC essential oils. It can be seen from Figure 2 that when the mass concentration of JH and SLC essential oil is 50 mg/L, it has a certain effect on the proliferation of THP-1 cells, and the cell morphology at this time has no obvious change from that before the treatment, and it is still bright and full. Therefore, the optimal concentration of JH and SLC essential oils on THP-1 was determined to be 50 mg/L, and subsequent efficacy screening tests were conducted with this mass concentration.

#### 3.4.2. Effects of JH and SLC Essential Oils on Proinflammatory Cytokines in Heat-Killed *P. acnes*-Treated THP-1 Cells

**Figure 3:**
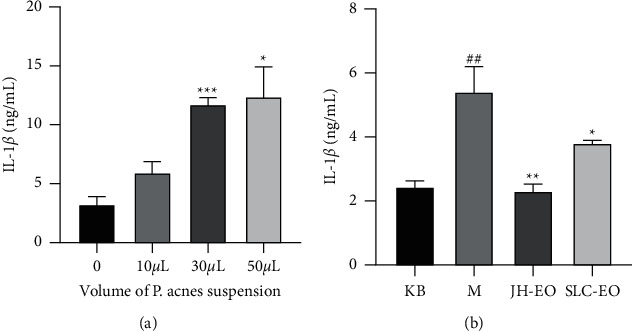
Influences of secretion of inflammatory cytokines IL-1*β* in THP-1 cells induced by *P. acnes* suspension. (a) Effect of different volume of *P. acnes* suspension on the secretion of IL-1*β*. (b) Effects of JH and SLC essential oils on secretion of IL-1*β*. The data are presented as the mean ± SD. ^*∗*^*p* < 0.01, ^*∗∗*^*p* < 0.01, ^*∗∗∗*^*p* < 0.001, and ^##^*p* < 0.01 versus the control group, by one-way ANOVA with Tukey's test.

We first examined how much volume (10 *μ*L, 30 *μ*L, and 50 *μ*L) of heat-killed *P. acnes* suspension was capable of inducing secreted inflammatory cytokines. The expression level of proinflammatory cytokines was measured by ELISA. As can be seen from Figure 3(a), when the liquid volume of *P. acnes* inactivated bacteria was 30 *μ*L, the inflammatory cytokines released were significantly different from those of the blank group, so 30 *μ*L *P. acnes* suspension was selected for the next experiment. Heat-killed *P. acnes* increased the secretion of IL-1*β* compared with the normal control (KB) in THP-1 cells. However, JH and SLC essential oils treatment significantly inhibited the secretion of cytokine. Thus, these observations suggest that JH and SLC essential oils effectively inhibit the secretion of IL-1*β* in THP-1 cells (Figure 3(b)). In addition, JH essential oil has a better effect than SLC essential oil in alleviating inflammation of THP-1 cells, which may be related to the high content of sesquiterpenes in JH essential oil [30, 31].

## 4. Conclusions

In this paper, a total of 124 compounds were obtained and 114 compounds were identified from 6 volatile oils sample of *C. morifolium*. The main constituents were terpenoids, heterospiroolefins, and esters compounds, including alkanes, fatty acids, phenols, alcohols, and aldehydes. Among them, the content of terpenoids in the essential oil of *C. morifolium* from different mature periods increased with the prolonging of flowering time, but the activity of the essential oil against *S. aureus* and *P. acnes* did not change significantly. However, the changes of chemical composition of the essential oils from different parts of *C. morifolium* had a great effect on the antibacterial activity, and the antibacterial activity was from high to low: JH > SLC > RC essential oil. Finally, the anti-inflammatory effects of JH and SLC essential oils were evaluated by cell experiments in vitro, which indicated that both JH and SLC essential oils had an alleviating effect on THP-1 cell inflammation induced by heat-killed *P. acnes* suspension. These findings, considered together, suggested that the oils from different flowering stages and nonmedicinal parts of *Chrysanthemum morifolium* Ramat. have certain antibacterial activity against *S. aureus* and *P. acnes* and it showed a good prospect in the development and application of anti-inflammatory and acne cosmetics.

## Figures and Tables

**Table 1:** Chemical composition (%) of JM, TJ, JH, SLC, and RC from *C. morifolium*.

Peak	Compound^a^	Content (%)^b^					RI^c^	RI^d^
		JM	TJ	JH	SLC	RC		
1	*cis*-1,3-Dimethylcyclohexane	0.32	0.15	0.04	—^e^	—	764	772
2	(*E*)-2-Hexenal	0.08	—	0.03	—	—	832	850
3	1,8-Cineole	—	0.11	—	—	—	1037	1031
4	*γ*-Terpinene	—	0.04	—	—	—	1062	1064
5	*cis*-Sabinol	—	0.07	0.04	—	—	1133	1139
6	Camphor	0.1	0.03	0.12	—	—	1144	1146
7	*cis*-Chrysanthenol	0.11	0.06	0.04	—	—	1156	1163
8	Borneol	1.17	1.13	0.37	0.22	—	1171	1172
9	Terpinen-4-ol	0.4	0.3	0.07	0.06	—	1185	1180
10	Fenchyl acetate	—	—	0.06	—	—	1221	1225
11	Carvacrol	2.33	2.09	0.4	0.47	—	1291	1291
12	*p*-Vinyl guaiacol	—	0.1	0.08	0.27	0.17	1314	1309
13	*α*-Copaene	—	—	—	0.2	—	1361	1369
14	*β*-Elemene	—	0.56	—	0.11	—	1385	1388
15	Italicene	0.14	0.13	—	0.15	0.09	1401	1406
16	Caryophyllene	1.24	1.2	0.09	8.84	0.1	1427	1428
17	*trans*-*α*-Bergamotene	—	—	—	0.13	—	1437	1430
18	(E)-*β*-Famesene	0.16	0.37	—	1.14	0.11	1455	1455
19	*α*-Caryophyllene	—	0.06	—	0.52	—	1462	1456
20	(+)-Spathulenol	2.15	2.97	0.15	—	—	1468	1472
21	7-epi-1,2-Dehydrosesquicineole	0.05	0.06	0.15	—	—	1484	1473
22	*β*-Selinene	0.87	1.09	0.34	—	0.05	1488	1485
23	*α*-Curcumene	1.82	3.63	—	2.69	—	1492	1485
24	Germacrene D	0.11	—	—	4.33	—	1494	1487
25	(Z,E)-*α*-Farnesene	1.68	0.12	0.36	0.21	—	1498	1495
26	Zingiberene	—	2.94	—	3.63	—	1502	1495
27	*α*-Muurolene	0.66	0.11	—	0.05	—	1504	1502
28	*α*-Farnesene	0.2	0.79	0.08	1.53	—	1510	1507
29	*γ*-Cadinene	—	0.26	—	0.4	—	1513	1516
30	*β*-Sesquiphellandrene	2.21	3.75	0.58	3.49	0.08	1523	1525
31	*γ*-Cadinene	0.3	0.32	0.09	—	—	1533	1534
32	(E)-*α*-Bisabolene	0.6	0.21	0.07	0.07	—	1540	1540
33	*α*-Calacorene	—	0.98	0.51	0.07	—	1543	1545
34	Selina-3,7(11)-diene	—	0.07	0.07	0.2	—	1562	1550
35	1-Formyl-2,2-dimethy-3-trans-(3-methyl-but-2-enyl)-6-Methyldene	—	0.14	0.26	0.06	—	1564	—
36	Germacrene B	—	—	0.65	—	—	1573	1579
37	Germacrene A	—	0.09	0.1	—	—	1577	1567
38	*trans*-Nerolidol	—	—	0.53	—	0.05	1581	1574
39	(-)-Spathulenol	0.14	0.23	1.48	0.22	—	1587	1585
40	1-Methylene-2B-hydroxymethyl-3,3-dimethyl-4B-(3-methylbut-2-enyl)	—	0.35	—	2.24	0.45	1591	—
41	Germacrene D-4-ol	—	0.43	0.63	—	—	1596	1597
42	Spathulenol	0.81	0.14	6.77	0.27	0.18	1599	1598
43	Caryophyllene oxide	—	2.81	1.04	—	—	1607	1603
44	Cedrol	—	0.34	0.37	—	—	1609	1609
45	Caryophyllen-5-ol	0.74	—	4.89	—	0.2	1614	1614
46	3-Cyclohexene-1-ethanol, *α*-ethenyl-*α*,3-dimethyl-6-(1-methylethylidene)-	—	1.7	—	—	0.35	1617	—
47	(*E*)-Sesquilavandulol	—	—	0.4	—	0.16	1619	1623
48	(+)-Junenol	—	—	4.56	—	0.12	1626	1627
49	*β*-Acorenol	1.2	3.75	1.71	—	0.06	1628	1630
50	*γ*-Eudesmol	—	—	2.48	—	—	1636	1637
51	Selin-6-en-4*α*-ol	0.33	0.71	0.37	0.28	—	1638	1636
52	*ζ*-Cadinol	0.14	0.19	2.15	—	9.21	1641	1645
53	*τ*-Muurolol	0.13	0.24	1.48	0.52	6.93	1645	1648
54	Selin-11-en-4-*α*-ol	—	0.21	—	—	2.1	1651	1654
55	epi-*α*-Muurolol	—	0.08	—	0.4	2.99	1657	1658
56	*α*-Cadinol	0.74	6.44	28.62	0.82	2.85	1659	1663
57	epi-*β*-Bisabolol	—	—	—	—	1.34	1666	1667
58	*β*-Cadinol	0.55	1.41	4.41	—	0.35	1668	1668
59	Cinnamic acid	—	—	0.14	0.06	0.86	1677	—
60	Torreyol	0.16	—	1.66	—	1.71	1679	1653
61	epi-*α*-Bisabolol	—	0.47	0.37	—	0.95	1684	1682
62	(*Z*)-*α*-trans-Bergamotol	0.14	0.55	1.22	0.53	0.14	1689	1686
63	5-Cyclodecen-1-ol	—	0.26	0.82	0.3	0.57	1695	1694
64	7-epi-*α*-Eudesmol	—	—	0.9	—	0.23	1705	—
65	*α*-Cyperone	—	0.07	0.75	—	1.42	1709	1706
66	14-Hydroxy-*α*-humulene	—	0.05	0.79	—	—	1716	1717
67	Farnesol	—	—	—	—	1.48	1726	1722
68	2-cis,6-cis-Farnesol	—	—	1.69	0.09	0.28	1729	1726
69	Curcumenol	—	0.04	0.23	0.06	0.09	1733	1736
70	Chamazulene	—	—	0.09	0.28	0.28	1739	1734
71	iso-Caryophyllene	0.61	—	1.77	—	—	1749	1737
72	1-Bisabolone	—	0.19	—	0.27	1.72	1751	1752
73	(*E*,*Z*)-Farnesol	—	—	0.1	—	0.51	1754	1749
74	(*R*)-(−)-Xanthorrhizol	—	—	—	2.03	1.62	1761	1753
75	Drimenol	—	—	—	—	0.85	1764	1770
76	3,4-Dimethoxyphenylacetic acid	—	—	0.11	0.1	0.76	1769	—
77	*β*-Sitosterol	0.25	—	0.43	0.1	—	1774	1774
78	*α*-Costol	0.18	0.06	1.39	0.51	0.38	1778	1778
79	Methyl-5,6,7,8-Tetrahydro-5-oxo-2-hydroxyquinolin-4-yl acetate	—	—	—	0.16	0.85	1786	—
80	Oplopenone isomer	0.08	—	0.14	0.37	1.02	1788	1784
81	(−)-Globulol	1.31	0.11	0.55	0.42	1.5	1794	1790
82	7-(2,4-Hexadiynylidene)-1,6-dioxaspiro[4,4]nona-2,8-diene	0.04	—	—	1.39	—	1798	1802
83	*n*-Octadecane	0.96	—	0.18	0.15	0.42	1801	1800
84	2-Naphthalenemethanol	0.45	—	—	2.07	—	1802	1805
85	Octadecanal	0.73	—	0.24	0.76	0.16	1817	1814
86	/^f^	0.16	—	—	0.59	—	1823	—
87	—	0.1	—	—	0.19	0.47	1830	—
88	2-Octenoic acid	0.25	—	0.37	—	0.12	1833	—
89	—	—	0.06	—	0.31	—	1838	—
90	Hexahydrofarnesyl acetone	1.97	0.06	0.46	0.13	—	1841	—
91	Ibuprofen	0.22	—	—	1.45	—	1847	—
92	Hexahydrofarnesyl acetone	—	—	—	0.39	—	1851	1846
93	2-trans,6-cis-Farnesol	0.38	—	—	—	—	1855	1844
94	2-trans,6-trans-Farnesyl acetate	0.04	—	0.2	6.53	32.00	1857	1854
95	Pentadecanoic acid	0.13	—	—	0.52	0.06	1865	1862
96	—	0.36	—	—	0.15	—	1871	—
97	Phthalic acid, diisobutyl ester	0.15	—	—	0.55	—	1876	1869
98	1-Hexadecanol	0.07	—	—	0.56	—	1887	1883
99	Palmitic acid, ethyl ester	—	—	—	0.42	—	1893	1993
100	Bicyclo[4.1.0]heptane, 7-pentyl-	—	—	—	0.64	—	1906	—
101	Epiglobulol	0.75	—	0.18	—	—	1917	—
102	(*Z*)-Tibetin spiroether	1.5	1.24	0.32	6.8	1.54	1920	1925
103	Palmitic acid methyl ester	0.48	0.11	—	—	0.12	1925	1925
104	Hexadecanoic acid, methyl ester	0.84	0.91	0.2	0.18	—	1930	1926
105	7-Isopropyl-1,4-dimethyl-2-azulenol		0.14	—	—	0.32	1944	1936
106	(*E*)-Tibetin spiroether	43.31	27.03	6.95	20.53	8.35	1954	1960
107	Dibutyl phthalate	0.54	0.6	0.76	0.13	—	1964	1968
108	*n*-Hexadecanoic acid	0.1	—	0.16	—	—	1985	1983
109	*n*-Eicosane	1.61	0.45	0.04	0.39	0.06	2001	2000
110	(*Z*)-Nuciferyl isovalerate	2.93	6.58	0.28	0.83	0.14	2025	2025
111	Manool	2.13	2.8	0.2	0.47	—	2037	2027
112	Methyl linoleate	0.21	0.2	0.08	0.4	0.38	2097	2094
113	Phytol	0.21	0.66	—	5.77	0.08	2113	2122
114	Methyl octadecanoate	0.6	0.39	0.09	0.08	0.21	2135	2124
115	Ethyl linolenate	0.04	0.03	—	0.25	—	2143	2159
116	Methyl linolenate	—	—	5.21	—	—	2227	2239
117	(*Z*)-Icos-9-en-1-ol	0.03	—	—	1.46	0.03	2250	2261
118	—	0.25	0.3	—	—	0.37	2289	—
119	Tricosane	0.7	1.06	0.92	0.93	0.1	2297	2300
120	2,2′-Methylene Bis (6-Tert-Butyl-4-Methyl)Phenol	—	0.05	0.07	—	—	2367	2365
121	—	—	0.57	0.77	—	—	2405	—
122	Heneicosanoic acid	2.68	—	—	—	—	2455	2463
123	*n*-pentaccosane	5.3	4.2	—	1.63	—	2496	2500
124	—	1.25	0.93	—	0.83	—	—	—
	Monoterpene hydrocarbons (%)	—	0.04	—	—	—		
	Oxygenated monoterpenes (%)	1.78	1.7	0.64	0.28	—		
	sesquiterpene hydrocarbons (%)	10.65	16.74	4.86	27.76	0.43		
	Oxygenated sesquiterpenes (%)	12.47	26.91	73.24	12.09	41.39		
	Fatty acids (%)	4.22	0.91	0.98	2.73	1.8		
	Hydrocarbons (%)	8.89	5.86	1.18	3.1	0.58		
	Esters (%)	2.02	1.3	6.4	7.69	32.71		
	Aldehydes (%)	0.81	—	0.27	0.76	0.16		
	Alcohols (%)	0.56	0.66	0.43	7.89	0.11		
	Phenols (%)	4.3	2.3	1.01	1.26	0.17		
	Heterospiroolefins (%)	44.81	28.27	7.27	27.33	9.89		
	Other (%)	3.05	6.58	0.42	3.39	2.01		
	Total identified (%)	93.56	91.27	96.7	94.28	89.25		
	No found (%)	2.12	1.86	0.77	2.07	0.84		
	Total (%)	95.68	93.13	97.47	96.35	90.09		
	Yield (%)	0.44	0.35	0.21	0.16	0.12		

^a^Compounds are listed according to their elution order on the apolar Elite-5MS capillary column. ^b^Contents (%) were calculated by electronic integration of peak areas obtained on the apolar Elite-5MS column. ^c^RI: Retention indices relatives to *n*-alkanes (C7–C27) on the apolar Elite-5MS column. ^d^RI: Retention indices relatives were obtained from the literature (19–23) and NIST Chemistry WebBook. ^e^−: not detected. ^f^/: not identified and not found.

**Table 2:** Antibacterial activities (MIC mg/mL) of six essential oils of *C. morifolium*.

Number	Samples	*Propionibacterium acacia* (ATCC6919)	*Staphylococcus aureus* (ATCC25923)
		MIC^a^ (mg/mL)	MIC (mg/mL)
1	JM	25	10
2	TJ	25	10
3	JH	25	10
4	SLC	25	20
5	RC	50	50
6	Solvent control	+^b^	+
7	Negative control	−^c^	−
8	Penicillin	/^d^	0.0035
9	Erythromycin lactobionate	0.061	—

^a^MIC: minimum inhibitory concentration. ^b^+: no bacteria grew, indicating that there were no other miscellaneous bacteria in the blank solvent and medium. ^c^−: there is bacterial growth, showing that the solvent has no inhibitory effect on bacteria. ^d^/: the minimum inhibitory concentration (MIC) of this bacterium was not tested.

## Data Availability

The datasets used during the present study are available from the corresponding author upon reasonable request.
